# Lisdexamfetamine maintenance treatment for binge-eating disorder following successful treatments: randomized double-blind placebo-controlled trial

**DOI:** 10.1017/S003329172400148X

**Published:** 2024-09

**Authors:** Carlos M. Grilo, Valentina Ivezaj, Sydney Yurkow, Cenk Tek, Ashley A. Wiedemann, Ralitza Gueorguieva

**Affiliations:** 1 Department of Psychiatry, Yale University School of Medicine, New Haven, CT, USA; 2 Department of Biostatistics, Yale School of Public Health, New Haven, CT, USA

**Keywords:** binge eating, cognitive-behavioral therapy, eating disorders, obesity, pharmacotherapy, weight loss

## Abstract

**Background:**

Controlled research examining maintenance treatments for responders to acute interventions for binge-eating disorder (BED) is limited. This study tested efficacy of lisdexamfetamine (LDX) maintenance treatment amongst acute responders.

**Methods:**

This prospective randomized double-blind placebo-controlled single-site trial, conducted March 2019 to September 2023, tested LDX as maintenance treatment for responders to acute treatments with LDX-alone or with cognitive-behavioral therapy (CBT + LDX) for BED with obesity. Sixty-one (83.6% women, mean age 44.3, mean BMI 36.1 kg/m^2^) acute responders were randomized to LDX (*N* = 32) or placebo (*N* = 29) for 12 weeks; 95.1% completed posttreatment assessments. Mixed-models and generalized-estimating equations comparing maintenance LDX *v.* placebo included main/interactive effects of acute (LDX or CBT + LDX) treatments to examine their predictive/moderating effects.

**Results:**

Relapse rates (to diagnosis-level binge-eating frequency) following maintenance treatments were 10.0% (*N* = 3/30) for LDX and 17.9% (*N* = 5/28) for placebo; intention-to-treat binge-eating remission rates were 59.4% (*N* = 19/32) and 65.5% (*N* = 19/29), respectively. Maintenance LDX and placebo did not differ significantly in binge-eating but differed in weight-loss and eating-disorder psychopathology. Maintenance LDX was associated with significant weight-loss (−2.3%) whereas placebo had significant weight-gain (+2.2%); LDX and placebo differed significantly in weight-change throughout treatment and at posttreatment. Eating-disorder psychopathology remained unchanged with LDX but increased significantly with placebo. Acute treatments did not significantly predict/moderate maintenance-treatment outcomes.

**Conclusions:**

Adults with BED/obesity who respond to acute lisdexamfetamine treatment (regardless of additionally receiving CBT) had good maintenance during subsequent 12-weeks. Maintenance lisdexamfetamine, relative to placebo, did not provide further benefit for binge-eating but was associated with significantly better eating-disorder psychopathology outcomes and greater weight-loss.

Binge-eating disorder (BED), a prevalent psychiatric disorder associated strongly with obesity and psychosocial/functional impairments, is characterized by high persistence/chronicity (Udo & Grilo, 2018). While there exists an evidence base for treatments with acute efficacy for BED (Grilo, Ivezaj, & Gueorguieva, 2024), controlled research examining maintenance treatments for responders to initial interventions is limited. This research gap is especially important for pharmacological approaches, which – in contrast to the longer-term durability of specific psychological treatments such as cognitive-behavioral therapy (Grilo, Masheb, Wilson, Gueorguieva, & White, 2011; Wilson, Wilfley, Agras, & Bryson, 2010) – are associated with high relapse rates following acute treatment (Grilo, Crosby, Wilson, & Masheb, 2012a).

Only two placebo-controlled trials have tested pharmacotherapy *maintenance* treatments for BED (Grilo, Lydecker, & Gueorguieva, 2023; Hudson, McElroy, Ferreira-Cornwell, Radewonuk, & Gasior, 2017). Hudson et al. (2017) found that continued lisdexamfetamine reduced relapse risk relative to placebo (3.7% *v.* 32.1%) in patients with BED who responded to acute treatment with lisdexamfetamine (Hudson et al., 2017). Grilo et al. (2023) found that naltrexone + bupropion following response to acute treatment with naltrexone + bupropion was associated with good maintenance of low binge-eating frequency and remission, and with significant additional weight loss. In contrast, maintenance medication (naltrexone + bupropion *v.* placebo) was not associated with any significant changes in binge-eating or weight in patients who responded to initial treatments with behavioral therapy (i.e. acute behavioral therapy – which showed good maintenance – significantly moderated the outcomes of maintenance pharmacotherapy).

This study was a prospective, randomized, double-blind, placebo-controlled test of the efficacy of lisdexamfetamine maintenance treatment following successful acute treatments with either lisdexamfetamine or lisdexamfetamine combined with cognitive-behavioral therapy (LDX *v.* CBT + LDX) for BED comorbid with obesity. This study logically examined maintenance pharmacotherapy following the ‘best-established’ (only FDA-approved) pharmacological (McElroy et al., 2015, 2016) and psychological (Grilo et al., 2024) treatments for BED. The present prospective controlled *maintenance* treatment study follows the *acute* treatment trial examining LDX *v.* CBT + LDX for BED comorbid with obesity for BED comorbid with obesity (Grilo et al., in press). Patients categorized as ‘responders’ following acute treatments were randomized, in double-blind fashion, with initial acute treatment (LDX alone or CBT + LDX) as a stratifying variable, to either maintenance LDX or placebo for 12 weeks without any additional psychotherapeutic interventions.

We hypothesized that participants receiving LDX would maintain reductions in binge-eating frequency and weight-loss attained with acute treatment while participants receiving placebo would show increases in binge-eating frequency and weight. We tested whether initial acute treatment with CBT predict or moderate outcomes of the maintenance treatment.

## Method

### Procedure

This single-site RCT was approved by the Yale institutional review board and included a data safety and monitoring plan with a physician safety monitor. Participants provided written informed consent.

### Participants

Participants for this controlled maintenance trial were eligible if they were categorized as ‘responders’ following initial acute 12-week treatments with LDX-alone or with CBT + LDX in a RCT for BED with obesity (Grilo et al., in press). When participants enrolled in the treatment study, they were informed and they consented to two treatment stages – i.e. the acute treatment stage (Grilo et al., in press) and a second stage that would test pharmacotherapy maintenance if they responded to the initial treatments comprising pharmacotherapy. Accordingly, participation in this maintenance treatment study was anticipated when participants consented to the 2-stage (initial acute ‘Stage 1’ plus maintenance ‘Stage 2’) treatments without an opt-out (i.e. if they responded to Stage 1, they participated in Stage 2, unless medically contraindicated).

Eligibility for the acute trial required meeting *DSM-5* (APA, 2013) BED criteria, ages 18–64 years old, and a body mass index (BMI; kg/m^2^) ≥30.0 and ≤50.0 (or ≥27.0 with obesity-related comorbidity). Exclusion criteria included clinical issues that required alternative treatments or represented contraindications to lisdexamfetamine, including: current evidence-based treatment for eating/weight disorders, taking contraindicated medications (e.g. opiates, MAOIs, NDRIs, and stimulants; SSRIs and strong inhibitors of CYP2D6 were considered on a case-by-case basis for safety/risk and depending on dosing), participation in another clinical trial/study, uncontrolled medical conditions, contraindications to LDX (e.g. alcohol and substance use disorders, psychosis/bipolar disorder, seizure history, cardiovascular disease, cerebrovascular disease, history renal, hepatic, or chronic pulmonary disease, systolic blood pressure > 160 mmHg, diastolic blood pressure > 100 mmHg, or heart rate > 100 beats/min), and pregnancy/breastfeeding.

#### Responder to acute treatment definition

Successful ‘response’ to the initial acute 12-week treatments was defined as ≥65% reduction (relative to baseline) in past-month frequency of binge-eating at posttreatment. This definition for response to treatment was used previously in the maintenance trial testing naltrexone + bupropion (Grilo et al., 2023) and in an adaptive stepped-care treatment trial to determine whether to continue treatment (for ‘responders’) or to switch to an alternative treatment in the case of ‘non-responders’ (Grilo et al., 2020). This definition was adopted based on several studies reporting that this cut-point, originally defined empirically using signal detection methods, consistently predicted treatment outcomes across both pharmacological and psychological treatments for BED (Grilo, Masheb, & Wilson, 2006; Grilo, White, Masheb, & Gueorguieva, 2015; Grilo, White, Wilson, Gueorguieva, & Masheb, 2012b).

Participants with ≥65% or greater reduction in binge-eating frequency were categorized as ‘responders’ and included in the present maintenance treatment trial. Binge-eating frequency (during previous month – i.e. 28 calendar days) was assessed using the Eating Disorder Examination Interview (EDE; Fairburn, Cooper, & O'Connor, 2008) at the posttreatment evaluation (Grilo et al., in press). The EDE was administered by doctoral-level assessors blinded to the treatment conditions. Posttreatment evaluation was performed immediately following completion of the initial acute treatments and eligible participants were randomized and began this maintenance treatment study within a week.

The 61 participants had mean age of 44.33 (s.d. = 10.86) years and mean BMI of 36.06 (s.d. = 4.49) kg/m^2^; 83.6% (*N* = 51) were female, 88.5% (*N* = 54) attended some/finished college, and 75.4% (*N* = 46) were White. Table 1 summarizes the participants' sociodemographic characteristics as well the specific Stage 1 treatments (LDX or CBT + LDX) received for the Stage 2 RCT participants, overall (*N* = 61) and separately for the LDX (*N* = 32) and placebo (*N* = 29) treatments.

**Table 1.** Demographic characteristics overall and across treatment conditions

	Overall	LDX	Placebo
	*N* = 61	*n* = 32	*n* = 29
Age, M (s.d.)	44.33 (10.86)	42.97 (11.38)	45.83 (10.24)
Sex (*n*, %)			
Male	10 (16.4)	5 (15.6)	5 (17.2)
Female	51 (83.6)	27 (84.4)	24 (82.8)
Race (*n*, %)			
White	46 (75.4)	25 (78.1)	21 (72.4)
Black	9 (14.8)	3 (9.4)	6 (20.7)
Asian	1 (1.6)	0 (0)	1 (3.4)
Multiracial	2 (3.3)	2 (6.3)	0 (0)
Other	3 (4.9)	2 (6.3)	1 (3.4)
Ethnicity (*n*, %)			
Hispanic or Latinx	12 (19.7)	8 (25.0)	4 (13.8)
Not Hispanic or Latinx	49 (80.3)	24 (75.0)	25 (86.2)
Sexual orientation (*n*, %)			
Heterosexual	58 (95.1)	29(90.6)	29 (100.0)
Gay or Lesbian	0 (0)	0 (0)	0 (0)
Bisexual	3 (4.9)	3 (9.4)	0 (0)
Other	0 (0)	0 (0)	0 (0)
Education (*n*, %)			
Up to high school	7 (11.5)	3 (9.4)	4 (13.8)
Some college	10 (16.4)	5 (15.6)	5 (17.2)
College	16 (26.2)	7 (21.9)	9 (31.0)
More than college	28 (45.9)	17 (53.1)	11 (37.9)
BMI, mean (s.d.)	36.06 (4.49)	35.42 (4.14)	36.76 (4.83)
Age onset BED, mean (s.d.)	26.66 (13.47)	23.97 (13.37)	29.62 (13.18)
Stage 1 acute treatment (*n*, %)			
LDX	29 (47.5)	15 (46.9)	14 (48.3)
LDX + CBT	32 (52.5)	17 (53.1)	15 (51.7)

*Note:* LDX, lisdexamfetamine; M, mean; s.d., standard deviation; *N*, number; BED, binge-eating disorder; CBT, cognitive behavioral therapy.

### Assessments

Assessment procedures were performed by trained/monitored doctoral-level research-clinicians who were independent from and blinded to treatments (both acute Stage 1 and maintenance Stage 2). The *Eating Disorder Examination* Interview (EDE; 16th-edition; Fairburn et al., 2008) was administered to assess binge-eating frequency and eating-disorder psychopathology (Global score) at baseline and posttreatment. The EDE has demonstrated good inter-rater and test–retest reliability in studies with BED (Grilo et al., 2022; Grilo, Masheb, Lozano-Blanco, & Barry, 2004). *Weight and height* were measured at baseline and weight was measured monthly and at post-treatment. When being weighed using digital scales, participants wore lightweight clothing without shoes.

To complement the primary interview measures at baseline and posttreatment, two self-report measures were administered monthly to capture data throughout the course of treatments. The *Eating Disorder Examination-Questionnaire* (Fairburn & Beglin, 1994), which has good test–retest reliability (Reas, Grilo, & Masheb, 2006), obtained binge-eating frequency and eating-disorder psychopathology (Global score) data during the past 28 days. The *Beck Depression Inventory-II* (Beck, Steer, & Brown, 1996) (alpha = 0.92) is well-established measure of depression symptoms/levels (Beck, Steet, & Garbin, 1998).

### Randomization and blinding procedures

The randomization schedule, developed by a biostatistician, assigned participants who received LDX treatment (i.e. either LDX or CBT + LDX) and were categorized as responders to the acute Stage 1 treatment to either LDX or placebo (double-blind) for 12-weeks. Responder status to Stage 1 acute treatments was defined as ≥65% reduction in binge-eating frequency. This cut-point for response was selected based on consistent findings regarding the positive prognostic significance of such reductions across both pharmacological and psychological treatments (Grilo et al., 2006, 2012b). This cut-point has been utilized in recent sequential designs (Grilo et al., 2020, 2023) and is supported by time course data for LDX (McElroy et al., 2017). ‘Responders’ were randomized in equal proportions to maintenance LDX or placebo, using stratified block randomization with Stage 1 acute treatment (LDX or CBT + LDX) as a stratifying variable. Assessors of outcomes were blinded to whether participants had received CBT during their prior Stage 1 treatment in addition to the (double-blind) medication in current trial.

### Treatments

Maintenance treatment involved solely the double-blind medication (LDX or placebo). There were no additional behavioral or psychotherapeutic interventions during this maintenance trial. At the end of Stage 1 acute treatment, down-titration of LDX occurred over a period of four days (50 mg for two days and 30 mg for two days). Stage 2 maintenance treatment began within a week of having completed Stage 1 acute treatment and having completed the down-titration. LDX treatment during this maintenance trial was administered following the dose-optimization (targeting 50–70 mg/day) protocol found superior to placebo (McElroy et al., 2016). During week 1, LDX was 30 mg/day for initial titration. During week 2, LDX was titrated to 50 mg/day. If the dose of 70 mg/day was not tolerated during the Stage 1 acute treatment, those participants were dosed at 50 mg/day for the remainder of Stage 2. If the dose of 70 mg/day was tolerated in Stage 1, during weeks 3–4, LDX was increased to 70 mg based on acceptable tolerability and clinical need. If patients developed intolerable side-effects on 70 mg in Stage 2, downward titration to 50 mg/day could occur, and if patients experienced adverse events and/or could not tolerate the medication, they were withdrawn from the medication. For the remaining treatment (weeks 3–12), the optimized LDX dose (50 or 70 mg/day) was maintained. Similar to Stage 1 acute treatment, down-titration consisted of four days (50 mg for two days and 30 mg for two days). Placebo was taken in capsules matched in appearance and frequency.

At the first (initial) maintenance trial study visit, study physicians delivered the pharmacotherapy, which focused on medication management (compliance, safety, and side-effects). Additional psychotherapeutic or behavioral interventions were proscribed. Monthly medication refills were accompanied by re-reviewing medication compliance and dosing schedules. Side-effect and safety checklists were performed monthly by research clinicians.

### Statistical analysis

Sample size for this maintenance trial was calculated for the comparison between LDX and placebo for maintenance (Hudson et al., 2017), based on estimated rates of response to acute treatments with LDX and CBT (Grilo et al., 2011; McElroy et al., 2016), and considering clinically meaningful effect-sizes. We estimated enrolling 80 participants would yield 64 with complete data at the end of Stage 2 which, in turn, would yield 84% power at two-sided alpha level of 0.05 to detect differences in relapse rates reported by Hudson et al. (2017) for LDX *v.* placebo (4% *v.* 32%). Our intent-to-treat sample (*N* = 61 total, with posttreatment data for *N* = 58) was only slightly lower than the target and thus we were well-powered (power of 80%) for a priori specified clinically meaningful effects.

Analyses comparing treatments were intention-to-treat, performed for all randomized patients who attended the first treatment session. Statistical testing was performed at 0.05 significance level and is reported with unadjusted *p*-levels.

The primary outcome variable was ‘relapse’ (to *DSM-5*-level of once-weekly binge-eating). Relapse was examined using two complementary ways (binge-eating frequency on EDE interview at posttreatment and EDE-Q monthly throughout).

The secondary outcome variables were binge eating, weight-loss, eating-disorder psychopathology, and depression. Binge eating was analyzed in two complementary ways – i.e. as a continuous variable (episode frequency monthly) and a categorical variable (binge-eating remission, defined as zero episodes in previous month (EDE-interview)) with missing data imputed as failure (i.e. non-remission). Remission was intended to complement the less stringent primary outcome relapse variable.

Weight (measured) was analyzed as a continuous outcome (percent weight-loss from start of maintenance trial). We further examined weight-loss as a categorical outcome (attaining ≥5% weight-loss during maintenance trial, with missing data imputed as failure) as ‘complementary’ analysis to percent weight-loss. Beyond intended ‘convergence,’ this categorical variable, a longstanding standard outcome in obesity trials (Greenway et al., 2010) and increasingly in BED trials (Grilo et al., 2022), is clinically useful as it is associated with cardiometabolic benefits in BED/obesity (Yurkow, Ivezaj, & Grilo, 2023).

For analyses of continuous variables, intention-to-treat analyses used all available data in mixed models without imputation. Variables not conforming to normality were log-transformed prior to analysis. Mixed models were constructed with fixed factors including maintenance medication treatment during stage 2 (LDX *v.* placebo), treatment during stage 1 (LDX *v.* CBT + LDX), time (all relevant time points), and all possible interactions. When interactions with stage 1 treatment were not significant, they were dropped from the models for parsimony. In each model we considered different error structures and selected the best-fitting structure based on the Schwarz' Bayesian Criterion. Tests of effect slices and focused comparisons of least square means were used to explain significant effects in the models.

For categorical variable outcomes, intention-to-treat analyses involved generalized estimating equations (GEE) models. Logit link function was used with binomial response distribution. Predictor variables were the same as in the mixed models above. If models encountered convergence problems with all possible interactions, interactions were dropped, and the model refitted until a model with no convergence issues was identified.

## Results

### Randomization and participant flow through treatment study

**Figure 1.**
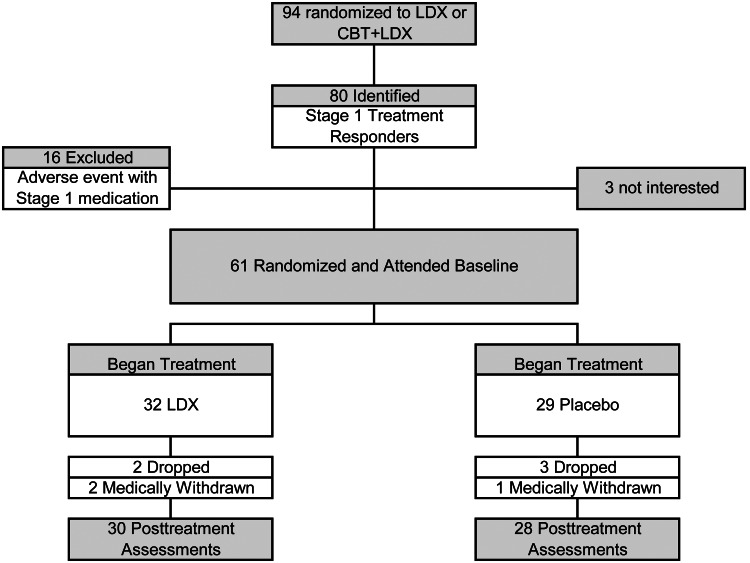
Participant flow throughout the study. Participant flow through this randomized double-blind controlled trial (RCT) testing lisdexamfetamine (LDX) *v.* placebo for *maintenance* treatment of patients with binge-eating disorder who responded successfully to acute treatments (Stage 1). Stage 1 treatment was a RCT testing LDX and cognitive-behavioral therapy (CBT) combined with LDX (i.e., CBT + LDX). Table 1 shows the specific Stage 1 treatments (a stratifying variable in the randomization) received by the participants in this Stage 2 maintenance RCT. Of the 94 participants randomized to LDX or CBT + LDX in Stage 1 treatment, 80 were categorized as treatment responders; of those, 16 were excluded due to adverse events during acute Stage 1 treatments and 3 were not interested in Stage 2 treatment. Thus, 61 were randomized and attended baseline treatment session for this RCT. Of the 61 participants in this Stage 2 maintenance RCT, 32 were randomized to LDX and 29 were randomized to Placebo. Overall, 5 (8.2%) dropped out and 3 (4.9%) were medically withdrawn (2 cases in LDX, one due to headaches and one due to dry eyes; 1 case in placebo due to increased blood pressure (See online Supplemental Table S1). Of the 61 participants, 58 (95.1%) completed posttreatment assessments.

Figure 1 (CONSORT) summarizes participant flow throughout the study. Of the 94 participants randomized to LDX or CBT + LDX in Stage 1 treatment, 80 were categorized as treatment responders; of those, 16 were excluded due to adverse events during acute Stage 1 treatments and 3 were not interested in Stage 2 treatment. Thus, 61 were randomized and attended baseline treatment session for this RCT. Of the 61, 32 were randomized to LDX and 29 were randomized to Placebo. Overall, 5 (8.2%) dropped out, 3 (4.9%) were medically withdrawn, and 58 (95.1%) completed posttreatment assessments.

### Primary outcomes

Relapse rates (meeting diagnosis-level binge-eating frequency determined with the EDE) following maintenance treatments (10.0% (*N* = 3/30) for LDX and 17.9% (*N* = 5/28) for placebo) did not differ significantly (χ^2^ (1) = 0.66, *p* = 0.42) nor were they associated with Stage 1 acute treatments (χ^2^ (1) = 0.85, *p* = 0.36). Two sensitivity analyses converged with this primary finding. First, because at baseline of this maintenance trial, 9.4% (*N* = 3/32) of those assigned to LDX and 0% (*N* = 0/29) of those assigned to placebo met diagnosis-level binge-eating frequency despite being ‘responders’ to acute treatment, sensitivity analysis testing relapse restricted to those who attained remission from binge eating was performed and it converged in indicating LDX and placebo did not differ (χ^2^ (1) = 1.26, *p* = 0.26). Second, a series of parallel analyses comparing relapse rates at each month (determined with the EDE-Q) throughout the course of maintenance treatments also revealed no significant differences between LDX and placebo.

### Secondary outcomes

**Figure 2.**
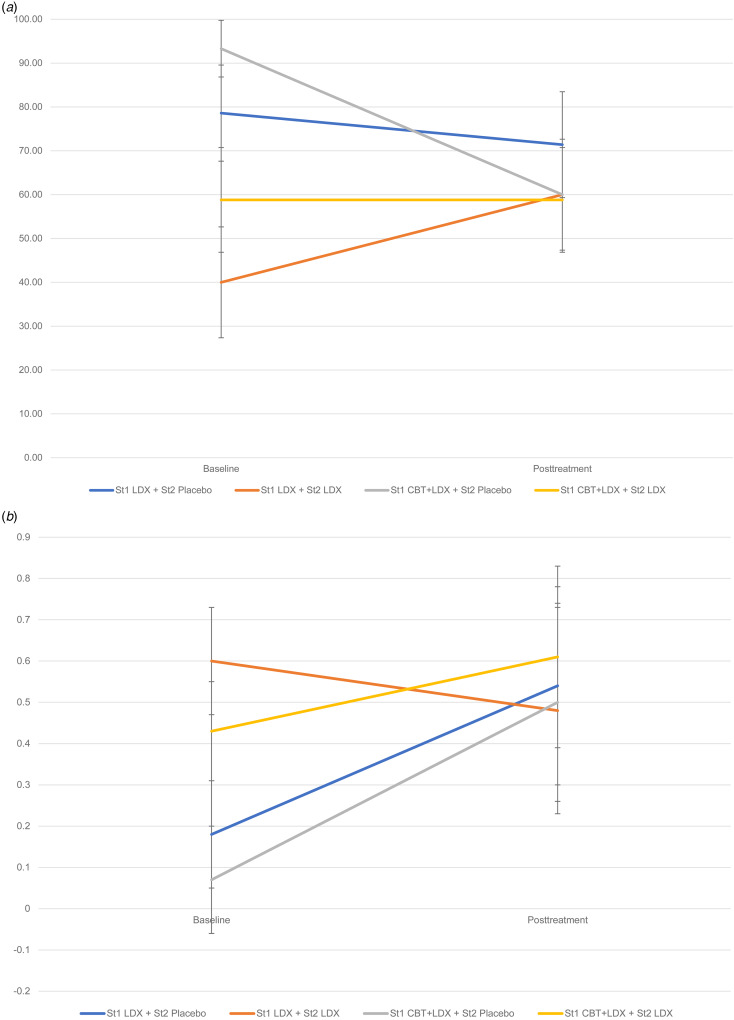
Binge-eating across the treatment medication conditions. 2a (top panel). Binge-eating remission rates during maintenance treatment (Stage 2) at baseline and at post-treatment. Remission rates are defined as zero episodes of binge eating during the last 28 days assessed using the Eating Disorder Examination Interview. The rates are based on the intention-to-treat sample (*N* = 61) with any missing data imputed as failure to remit. St1 = Stage 1 (acute treatment); St2 = Stage 2 (maintenance treatment). The four lines show the rates of binge-eating remission separately for lisdexamfetamine (LDX) and placebo conditions during maintenance (Stage 2) treatment separately by LDX or cognitive-behavioral therapy plus LDX (i.e. CBT + LDX) during initial acute (Stage 1) treatments. There were no significant interaction effects between the acute (Stage 1) treatment condition (LDX and CBT + LDX) and maintenance (Stage 2) treatments (LDX and placebo). Maintenance LDX and placebo did not differ significantly. 2b (bottom panel). Least Square Means (LSM) for frequency of binge-eating episodes during the past 28 days for the maintenance treatment (Stage 2) at baseline and at post-treatment (assessed using the Eating Disorder Examination interview). St1 = Stage 1 (acute treatment); St2 = Stage 2 (maintenance treatment). The four lines show the LSMs (error bars indicate standard errors) of binge-eating episode frequency separately for LDX and placebo conditions during maintenance (Stage 2) treatment separately by LDX or CBT + LDX during initial acute (Stage 1) treatments. There were no significant interaction effects between the acute (Stage 1) treatment condition (LDX and CBT + LDX) and maintenance (Stage 2) treatments (LDX and placebo). Maintenance LDX and placebo did not differ significantly.

**Figure 3.**
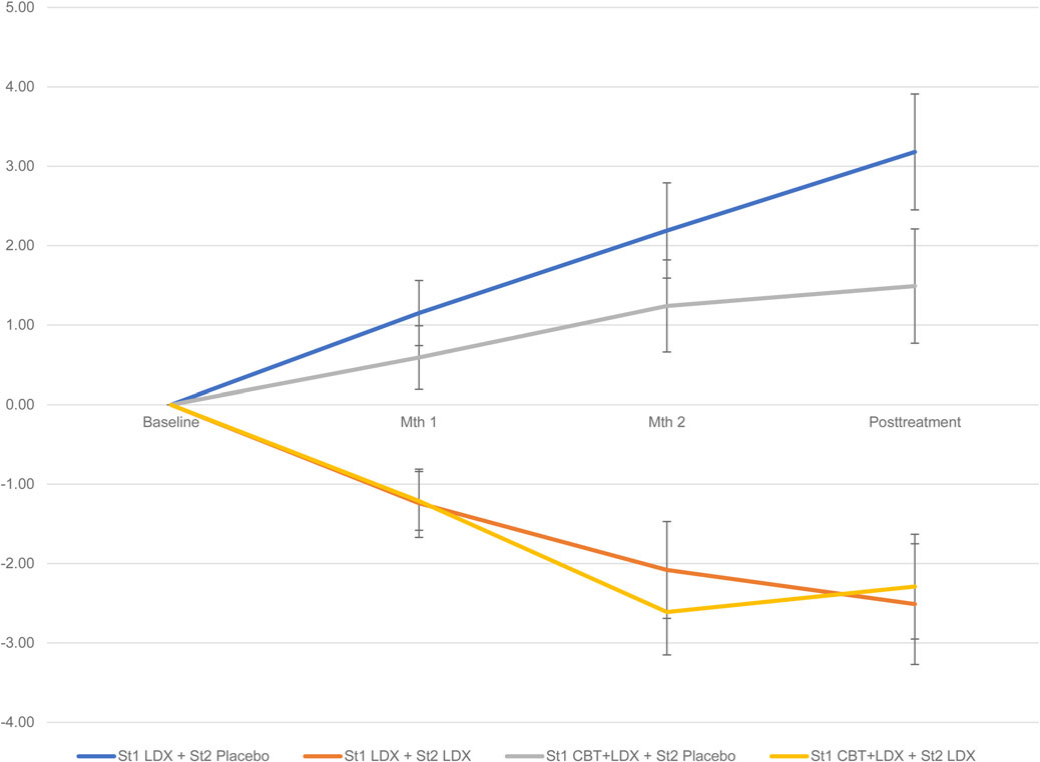
Percent weight loss across the treatment medication conditions. Least Square Means (LSM) of percent weight loss (from baseline start of maintenance treatment) calculated using measured values at baseline, measured monthly during maintenance treatment, and measured at post-treatment. St1 = Stage 1 (acute treatment); St2 = Stage 2 (maintenance treatment); Mth = Month. The four lines show the LSMs (error bars indicate standard errors) for lisdexamfetamine (LDX) and placebo conditions during maintenance (Stage 2) treatment separately by LDX or cognitive-behavioral therapy plus LDX (i.e. CBT + LDX) during initial acute (Stage 1) treatments. Analyses revealed significant interaction between maintenance treatment and time (*p* = 0.0002) and a significant main effect of maintenance treatment (*p* < 0.0001); acute (stage 1) treatment effects were not significant (*p* = 0.43). LDX maintenance had significant and progressive weight loss on average whereas placebo maintenance had significant and progressive weight gain on average. LDX and placebo were significantly different from each other at every monthly time-point through posttreatment (*p* < 0.0001).

The secondary outcome variables were binge eating, weight-loss, eating-disorder psychopathology, and depression. Table 2 shows descriptive statistics for secondary continuous outcomes and Figs 2 and 3 summarize statistical findings. There were no statistically significant effects for depression (BDI-II) scores; statistical findings for other outcomes are below.

**Table 2.** Clinical measures across treatment conditions

	LDX			Placebo		
	*n* = 32			*n* = 29		
	*n*	M	s.d.	*n*	M	s.d.
EDE Binge Eating						
Pre-treatment	32	1.00	1.39	29	0.21	0.56
Post-treatment	30	2.37	6.32	28	1.82	4.30
*Change*	30	1.33	5.82	28	1.61	4.37
EDE-Q Binge Eating						
Pre-treatment	31	2.26	3.18	29	0.76	1.70
Post-treatment	25	1.52	2.52	28	3.14	3.61
*Change*	25	−0.80	2.50	28	2.36	3.88
Weight						
Pre-treatment	32	222.94	40.81	29	225.83	38.48
Post-treatment	30	214.27	38.75	28	231.58	38.23
*Change*	30	−5.23	6.47	28	4.81	6.27
*% Change*	30	−2.39	2.95	28	2.25	2.51
EDE Global Score						
Pre-treatment	31	1.45	0.90	28	1.09	0.73
Post-treatment	29	1.39	1.08	28	1.59	0.88
*Change*	28	−0.05	0.77	27	0.50	0.64
EDE-Q Global Score						
Pre-treatment	31	2.12	1.14	29	1.62	1.02
Post-treatment	25	2.03	1.29	28	2.43	1.01
*Change*	25	−0.12	0.64	28	0.79	0.77
BDI-II Depression Score						
Pre-treatment	31	7.19	6.72	29	5.93	5.45
Post-treatment	25	7.76	7.71	28	6.18	7.05
*Change*	25	2.16	5.27	28	0.18	7.07

*Note:* EDE, Eating Disorder Examination Interview; EDE-Q, Eating Disorder Examination Examination–Questionnaire; BDI-II, Beck Depression Inventory-II; LDX, lisdexamfetamine; *N*, number; M, mean; s.d., standard deviation.

#### Binge-eating outcomes

Intention-to-treat binge-eating *remission rates* following maintenance treatments were 59.4% (*N* = 19/32) for LDX and 65.5% (*N* = 19/29) for placebo. Figure 2a illustrates remission rates did not differ significantly (χ^2^ (1) = 0.24, *p* = 0.62) nor were they associated with Stage 1 acute treatments (χ^2^ (1) = 0.24, *p* = 0.62). Sensitivity analysis restricted to completers revealed convergent non-significant findings.

Mixed models of *binge-eating frequency* during the past month at posttreatment (based on the EDE) revealed no significant effects, including no significant treatment-by-time effects (*F*(1,56.5) = 2.08, *p* = 0.15). Figure 2b illustrates binge-eating frequency findings showing maintenance LDX and placebo conditions did not differ significantly (*F*(1,57.9) = 2.13, *p* = 0.15) nor were they associated with Stage 1 acute treatments (*F*(1,58.2) = 0.91, *p* = 0.34).

Mixed models of *binge-eating frequency assessed monthly throughout the course of maintenance treatment* revealed a statistically significant interaction between maintenance treatment and time (*F*(3157) = 8.32, *p* < 0.0001) and a statistically significant main effect of acute (Stage 1) treatment (*F*(1,57.7) = 5.72, *p* = 0.02). Stage 1 LDX-only, relative to CBT + LDX, had higher overall means of binge-eating frequency throughout Stage 2 maintenance. During Stage 2 maintenance trial, binge-eating frequency increased significantly with placebo maintenance (*F*(3155) = 9.39, *p* < 0.0001) and decreased non-significantly with LDX maintenance (*F*(3158) = 1.30, *p* = 0.28). The placebo maintenance group, which had significantly lower binge-eating frequency at baseline (*F*(1135) = 6.69, *p* = 0.01), had significantly higher frequency than the LDX maintenance group at posttreatment (*F*(1147) = 4.57, *p* = 0.03).

#### Percent weight loss

Table 2 shows weight values at baseline and post-treatment and changes. Mixed models of *percent weight-loss at posttreatment* during the maintenance trial revealed a significant main effect of maintenance treatment (*F*(1,55) = 40.19, *p* < 0.0001); acute stage 1 treatment was not significant (*F*(1,55) = 1.22, *p* = 0.27). The LDX group had significant weight-loss (−2.33%) whereas the placebo group had significant weight-gain (+2.24%) on average (both *p* < 0.0001).

Figure 3 summarizes percent weight-losses for the LDX and placebo treatments throughout the course of the maintenance trial shown separately for those who received LDX or CBT + LDX during the acute (Stage 1) treatments. Mixed models of percent weight-loss monthly during the maintenance trial revealed a significant interaction between maintenance treatment and time (*F*(2,53.8) = 9.90, *p* = 0.0002) and a significant main effect of maintenance treatment (*F*(1,53.5) = 53.08, *p* < 0.0001); acute (stage 1) treatment was not significant (*F*(1,53) = 0.63, *p* = 0.43). The LDX group had significant and progressive weight loss on average whereas the placebo group had significant and progressive weight gain on average. LDX and placebo were significantly different from each other at every monthly time-point through posttreatment (*p* < 0.0001).

*Attaining 5% weight loss*. The group receiving LDX maintenance treatment was significantly more likely than the placebo group to attain ≥5% weight loss specifically during the maintenance period (18.8% (*N* = 6/32) *v.* 0% (*N* = 0/29); Fisher's exact test value = 0.025).

#### Eating-disorder psychopathology

**Figure 4.**
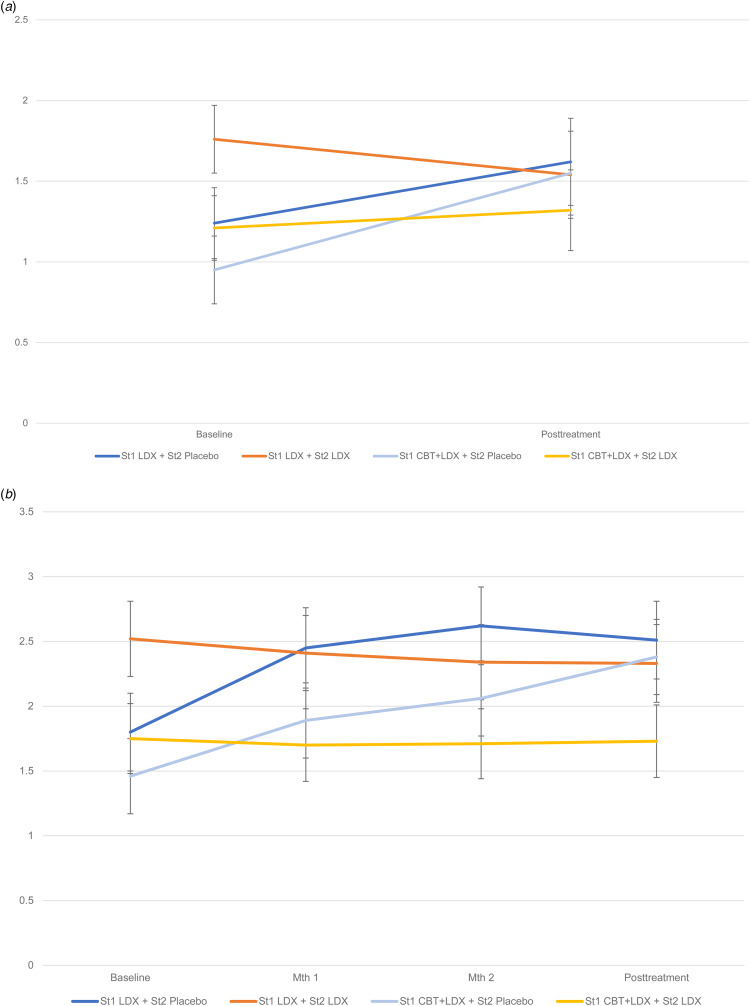
Eating-disorder psychopathology across treatment medication conditions. 4a (top panel). Least Square Means (LSMs) for global score on the Eating Disorder Examination Interview during maintenance treatment (Stage 2) at baseline and at post-treatment. St1 = Stage 1 (acute treatment); St2 = Stage 2 (maintenance treatment). The four lines show the eating-disorder psychopathology global scores (LSMs) separately for lisdexamfetamine (LDX) and placebo conditions during maintenance (Stage 2) treatment separately by LDX or cognitive-behavioral therapy plus LDX (i.e. CBT + LDX) during initial acute (Stage 1) treatments. Analyses revealed a statistically significant interaction between maintenance treatment and time (*p* = .01) and a statistically significant main time effect (*p* = 0.02). EDE global score increased significantly with placebo maintenance (*p* = 0.0005) whereas it did not change significantly with LDX maintenance (*p* = 0.79). 4b (bottom panel). Least Square Means (LSMs) for global score on the Eating Disorder Examination-Questionnaire at monthly assessments throughout the maintenance treatment (Stage 2) at baseline and at post-treatment. The four lines show the eating-disorder psychopathology global scores (LSMs) separately for lisdexamfetamine (LDX) and placebo conditions during maintenance (Stage 2) treatment separately by LDX or cognitive-behavioral therapy plus LDX (i.e. CBT + LDX) during initial acute (Stage 1) treatments. St1 = Stage 1 (acute treatment); St2 = Stage 2 (maintenance treatment).

Table 2 shows the eating-disorder psychopathology global score values at baseline and at posttreatment and changes separately for the EDE interview and EDE-Q are shown in Figure 4. Mixed models of EDE global score revealed a statistically significant interaction between maintenance treatment and time (*F*(1,54.9) = 7.88, *p* = 0.01) and a statistically significant main effect of time (*F*(1,54.9) = 5.95, *p* = 0.02). EDE global score increased significantly with placebo maintenance (*F*(1,54.9) = 13.51, *p* = 0.0005) whereas it did not change significantly with LDX maintenance (*F*(1,55) = 0.07, *p* = 0.79).

Mixed models of the EDE-Q global score monthly during the maintenance trial revealed a significant interaction between maintenance treatment and time (*F*(3154) = 10.83, *p* < 0.0001) and a significant main effect of time (*F*(3154) = 6.58, *p* = 0.0003); additionally, a statistically significant main effect of acute Stage 1 treatment was observed (*F*(1,58) = 4.25, *p* = 0.04) reflecting higher scores on average for those who received LDX than CBT + LDX during initial acute treatment. Throughout the maintenance trial, placebo had significant increase in EDE-Q Global scores (*F*(3154) = 17.28, *p* < 0.0001) whereas no significant changes occurred with LDX (*F*(3155) = 0.27, *p* = 0.85).

## Discussion

This prospective controlled *maintenance* treatment study of adults with BED with comorbid obesity – only the third controlled maintenance trial performed to date with BED – tested the effectiveness of LDX for maintenance following successful acute treatments with LDX (i.e. LDX-alone or combined with CBT). Participants with good outcomes following a controlled *acute* trial (Grilo et al., in press) were re-randomized, in double-blind fashion, to either LDX or to placebo for 12 weeks. Findings indicate that adults with BED with obesity with good responses to initial acute treatment with LDX – regardless of whether also having received CBT – showed good maintenance during the subsequent 12-weeks. Relapse (to diagnosis-level binge-eating frequency) was infrequent and did not differ between LDX and placebo. Intention-to-treat binge-eating remission rates following the maintenance trial were 59.4% for LDX and 65.5% for placebo. Importantly, although maintenance LDX, relative to placebo, did not provide further benefit for most measures of binge-eating, it was associated with significantly with better eating-disorder psychopathology outcomes and with greater further weight-loss.

Our findings supporting LDX *maintenance* treatment add to those previously reported by Hudson et al. (2017). Whereas Hudson et al. (2017) found that maintenance LDX reduced relapse risk relative to placebo (3.7% *v.* 32.1%), we did not find a significant difference (i.e. 10.0% *v.* 17.9%, respectively). The maintenance trial by Hudson et al. (2017) was 26 weeks (*v.* 12 weeks in the present trial) and that longer time-period could have allowed for more relapses to occur over time. However, while our shorter maintenance trial found no statistical advantage for maintenance LDX *v.* placebo for binge-eating (various categorical and continuous measures), LDX was significantly superior to placebo for maintaining improvements in associated eating-disorder psychopathology. Specifically, eating-disorder psychopathology remained unchanged with maintenance LDX but increased significantly with placebo. This finding may be clinically important in light of recent findings that certain aspects of associated eating-disorder psychopathology at posttreatment prospectively predicted relapse one year later in patients who had achieved remission from binge eating with behaviorally based treatments for BED (Grilo et al., 2024).

Contrary to hypothesis, having received CBT during the initial acute treatment (i.e. in addition to LDX) did not significantly moderate LDX maintenance treatment outcomes. Broadly, these findings contrast with those reported by Grilo et al. (2024) in a maintenance trial testing naltrexone/bupropion; in that trial, placebo was associated with worsening outcomes amongst those treated initially with naltrexone/bupropion but not amongst those who received behavioral therapy. The reasons for this are uncertain; we can speculate that the relatively brief duration of the maintenance trial might not have allowed enough time for additional binge-eating relapse events to occur following the high response and remission rates attained with the acute treatments.

Maintenance LDX was associated with significantly greater weight-loss (2.3% loss) than placebo (which had 2.2% gain); 18.8% of the LDX group attained 5≥% weight-loss during the maintenance trial. LDX treatments resulted in significant weight-loss during the initial acute trial of roughly 5% and the maintenance LDX treatment resulted in a further weight-loss (roughly 2.3%). These findings are notable as most psychological and pharmacological (except topiramate) trials for BED have reported little-to-no weight loss (Grilo et al., 2024) and behavioral weight-loss trials for BED have reported attenuated weight-losses in BED compared those reported for patients with obesity without BED (Forman et al., 2023). BED is associated with subsequent onsets of chronic cardiometabolic conditions (Hudson et al., 2010; Kessler et al., 2013) and research has found that modest weight losses (>5%) are associated with significantly improved glycemic control and reduced cardiometabolic abnormalities in patients with obesity (Wing et al., 2011) including those specifically with BED (Yurkow et al., 2023).

As context for the findings, we note methodological strengths and limitations. Methodological strengths include the randomized double-blind design, pharmacotherapy delivery by faculty-level physician without any additional psychotherapeutic or nutrition interventions, assessments performed by independent and blinded doctoral evaluators using well-validated measures, and high retention rates. Further, the study design allowed for prospective examination of potential moderating effects of CBT received during initial acute pharmacotherapy treatment. Several potential limitations are noteworthy. The sample size had limited power to detect smaller magnitude main/interactive effects of treatments. Generalizability of findings to different clinical settings, different providers, and to persons with BED without obesity, different sociodemographic and clinical profiles is uncertain. The participant group was 84% female, well-educated (roughly 72% attended some college), and had reasonable racial/ethnic representation (15% Black and 20% Hispanic).

Lastly, we note this maintenance trial was brief (only 12 weeks) and longer-term outcomes are unknown. With this important caveat highlighted, we cautiously comment on what practitioners and their patients can do after 12-week LDX maintenance treatment. Hudson et al. (2017), in their controlled maintenance trial for BED (double-blind randomized withdrawal design), found that LDX was associated with significantly decreased risk of relapse over a 26-week period alongside a well-tolerated and well-known side-effect profile for this medication. Thus, it seems reasonable for practitioners to discuss with their patients with BED with co-existing obesity who have benefitted from acute and maintenance trials with LDX and have no side-effects that continuing with the medication longer may be worth considering. In addition to reducing risk for relapse (Hudson et al., 2017), our study found that continued LDX was associated with significantly better eating-disorder psychopathology outcomes and greater weight-loss. We emphasize that continued or longer-term maintenance treatment with LDX should include careful on-going medical monitoring to ensure safety, address any emergent side-effects, identify any problematic medication misuse behaviors, and to identify any excessive weight-loss or emergence of eating-disorder psychopathology. We also highlight that our study group comprised participants carefully assessed to establish the absence of histories of alcohol/substance use disorders (given the US FDA's product labelling for LDX, which includes a ‘black box’ warning regarding potential for misuse) in addition to various important contraindications, including cardiac conditions. We also note that the US FDA labeling includes a ‘limitation of use’ that LDX is not indicated for weight loss and that its safety and efficacy for obesity are unknown. Thus, the discussion with patients regarding potential benefits/risks of LDX should also consider carefully the US FDA's product labelling alongside our study's outcomes and other medical data as they become available.

With these methodological considerations as context, we conclude that adults with BED with obesity who respond to acute (12-week) LDX (regardless of having additionally received CBT) could be offered maintenance treatment with LDX. Maintenance LDX, relative to placebo, did not provide further benefit for binge-eating but was associated with significantly better eating-disorder psychopathology outcomes and further weight-loss.

## Supporting information

Grilo et al. supplementary material Grilo et al. supplementary material

## Data Availability

De-identified data will be provided in response to reasonable written request to achieve goals in an approved written proposal.

## Supplementary material

The supplementary material for this article can be found at https://doi.org/10.1017/S003329172400148X.

## Clinicaltrials.gov registration: NCT03926052

(Cognitive-Behavioral and Pharmacologic (LDX) Treatment of Binge-Eating Disorder and Obesity: Maintenance Treatment)
