# Supplementary material for: Lisdexamfetamine maintenance treatment for binge-eating disorder following successful treatments: randomized double-blind placebo-controlled trial
Source: Psychol Med. 2024 Sep 11;54(12):3334–44. doi: 10.1017/S003329172400148X (PMC11496227; doi:10.1017/S003329172400148X)
Supplement: Grilo et al. supplementary material [file S003329172400148Xsup001.docx]

**SUPPLEMENTAL TABLE 1.** Adverse Events (frequency) during maintenance trial testing lisdexamfetamine and placebo treatments.

|  | **Lisdexamfetamine**  *n*=32  n (%) | **Placebo**  *n*=29  n (%) |
| --- | --- | --- |
| All-Cause Mortality | 0 (0.0%) | 0 (0.0%) |
| Serious Adverse Events 0 (0.0%) 0 (0.0%)  Adverse Events Leading to Discontinuation* 2 (6.3%) 1 (3.4%)  Adverse Events (not including Serious)** | | |
| Decreased Appetite | 10 (31.3%) | 4 (13.8%) |
| Dry Mouth | 6 (18.8%) | 1 (3.4%) |
| Insomnia | 6 (18.8%) | 4 (13.8%) |
| Increased Energy | 5 (15.6%) | 3 (10.3%) |
| Constipation | 3 (9.4%) | 1 (3.4%) |
| Jittery | 2 (6.3%) | 0 (0.0%) |
| Increased Heart Rate | 2 (6.3%) | 1 (3.4%) |

Note: Adverse (side effect) events assessed systematically at month one of maintenance randomized double-blind placebo-controlled trial testing lisdexamfetamine versus placebo. These adverse event data are included in clinicaltrials.gov record (NCT03926052).

* Adverse events leading to discontinuation (medical withdrawal) were reported for 2 cases in lisdexamfetamine (headache, dry eyes) and for 1 case in placebo (increased blood pressure).

** Adverse events reported by >5% of participants in lisdexamfetamine or placebo treatment condition.
